# Classifying MCI Subtypes in Community-Dwelling Elderly Using Cross-Sectional and Longitudinal MRI-Based Biomarkers

**DOI:** 10.3389/fnagi.2017.00309

**Published:** 2017-09-26

**Authors:** Hao Guan, Tao Liu, Jiyang Jiang, Dacheng Tao, Jicong Zhang, Haijun Niu, Wanlin Zhu, Yilong Wang, Jian Cheng, Nicole A. Kochan, Henry Brodaty, Perminder Sachdev, Wei Wen

**Affiliations:** ^1^School of Biological Science and Medical Engineering, Beihang University, Beijing, China; ^2^Beijing Advanced Innovation Center for Big Data-Based Precision Medicine, Beijing, China; ^3^Beijing Advanced Innovation Center for Biomedical Engineering, Beijing, China; ^4^Centre for Healthy Brain Ageing, School of Psychiatry, University of New South Wales, Sydney, NSW, Australia; ^5^Neuropsychiatric Institute, Prince of Wales Hospital, Sydney, NSW, Australia; ^6^UBTech Sydney Artificial Intelligence Institute, Faculty of Engineering and Information Technologies, University of Sydney, Darlington, NSW, Australia; ^7^The School of Information Technologies, Faculty of Engineering and Information Technologies, University of Sydney, Darlington, NSW, Australia; ^8^Beijing Tiantan Hospital, Capital Medical University, Beijing, China; ^9^NIBIB, NICHD, National Institutes of Health, Bethesda, MD, United States; ^10^Dementia Collaborative Research Centre, University of New South Wales, Sydney, NSW, Australia

**Keywords:** mild cognitive impairment, longitudinal data, early diagnosis, MRI, biomarker, feature selection, machine learning

## Abstract

Amnestic MCI (aMCI) and non-amnestic MCI (naMCI) are considered to differ in etiology and outcome. Accurately classifying MCI into meaningful subtypes would enable early intervention with targeted treatment. In this study, we employed structural magnetic resonance imaging (MRI) for MCI subtype classification. This was carried out in a sample of 184 community-dwelling individuals (aged 73–85 years). Cortical surface based measurements were computed from longitudinal and cross-sectional scans. By introducing a feature selection algorithm, we identified a set of discriminative features, and further investigated the temporal patterns of these features. A voting classifier was trained and evaluated via 10 iterations of cross-validation. The best classification accuracies achieved were: 77% (naMCI vs. aMCI), 81% (aMCI vs. cognitively normal (CN)) and 70% (naMCI vs. CN). The best results for differentiating aMCI from naMCI were achieved with baseline features. Hippocampus, amygdala and frontal pole were found to be most discriminative for classifying MCI subtypes. Additionally, we observed the dynamics of classification of several MRI biomarkers. Learning the dynamics of atrophy may aid in the development of better biomarkers, as it may track the progression of cognitive impairment.

## Introduction

Mild cognitive impairment (MCI) is thought to be a transitional stage between cognitively normal and dementia (Petersen, 2004). Previous studies have shown that neuroimaging biomarkers are potential predictors of cognitive impairment (Shi et al., 2010; Cuingnet et al., 2011; Davatzikos et al., 2011; Falahati et al., 2014; Trzepacz et al., 2014; Bron et al., 2015; Jung et al., 2016; Lebedeva et al., 2017). Many researchers have developed and implemented machine learning systems which use neuroimaging biomarkers for more accurate identification of individuals with MCI or dementia (Cui et al., 2012a; Shao et al., 2012; Lebedev et al., 2014; Min et al., 2014; Moradi et al., 2015; Yun et al., 2015; Cai et al., 2017; Guo et al., 2017). Early diagnosis is an essential step in the prevention and early treatment of MCI and dementia.

MCI is clinically heterogeneous with different risks of progression to dementia. Clinical subtypes of MCI have been proposed to broaden the concept, and included prodromal forms of a variety of dementias (Petersen, 2004). MCI is termed “amnestic MCI” (aMCI) when memory loss is the predominant symptom. Almost 10% to 15% aMCI individuals tend to progress to clinically probable Alzheimer's disease (AD) annually (Grundman et al., 2004). Additionally, MCI is termed “non-amnestic MCI” (naMCI) when impairments are in domains other than memory. Individuals with naMCI were more likely to convert to dementia other than AD, such as vascular dementia or dementia with Lewy bodies (Tabert et al., 2006). The progression of different MCI subtypes to a particular type of dementia has yet to be clearly delineated. On the other hand, MCI does not necessarily lead to dementia, since some studies suggested that MCI subjects have higher rates of reversion to normal cognition than progression to dementia (Brodaty et al., 2013; Pandya et al., 2016). A population-based study found that the reversion rate is lower in aMCI compared with naMCI (Roberts et al., 2014). Reliably identifying MCI of different subtypes would enable more efficient clinical trials and facilitate better targeted treatments.

Longitudinal measurements of Magnetic Resonance Imaging (MRI) in MCI and dementia may provide crucial predictors for tracking the disease progression of dementia (Misra et al., 2009; Risacher et al., 2010; Liu et al., 2013; Mayo et al., 2017). However, only a few studies used longitudinal data for automated classification of MCI and dementia (McEvoy et al., 2011; Li et al., 2012; Zhang et al., 2012a; Ardekani et al., 2017; Huang et al., 2017). Zhang et al. proposed an AD prediction method using longitudinal data which achieved greater classification results than using baseline visit data (Zhang et al., 2012a). Huang et al. presented a longitudinal measurement of MCI brain images and a hierarchical classification method for AD prediction. Their method using longitudinal data consistently outperformed the method using baseline data only (Huang et al., 2017). Despite these efforts, employing machine learning technique with longitudinal MRI features for MCI subtypes classification is rarely studied. And an additional aspect of research when using longitudinal MRI measurements is to identify the biomarkers that remain significant during the time course.

In this study, we used machine learning technique to classify MCI subtypes by employing cross-sectional and longitudinal MRI features. We reported nine independent classification experiments, whereby we compared two groups in each experiment: aMCI vs. cognitively normal (CN), naMCI vs. CN, naMCI vs. aMCI, using features measured at baseline, two-year follow-up, and longitudinally. The longitudinal features were employed by calculating the means and changes of the cross-sectional measurements. Clinical classifications at two-year follow-up were used as the comparison. The features used for classification were cortical surface based, including sulcal width, cortical thickness, cortical gray matter (GM) volume, subcortical volumes and white matter hyper-intensity (WMH) volume. We compared the classification performance using cross-sectional features and longitudinal features. In addition, we performed feature selection and analyzed the temporal patterns of the selected biomarkers.

## Materials and methods

### Participants

Participants were members of the Sydney Memory and Aging Study (MAS), a longitudinal study of community-dwelling individuals aged 70–90 years recruited via the electoral roll from two regions of Sydney, Australia (Sachdev et al., 2010). Individuals were excluded at baseline if they had a previous diagnosis of dementia, mental retardation, psychotic disorder including schizophrenia or bipolar disorder, multiple sclerosis, motor neuron disease, developmental disability, or progressive malignancy. The study was approved by the Ethics Committees of the University of New South Wales and the South Eastern Sydney and Illawarra Area Health Service. Written informed consent was obtained from each participant.

### Diagnosis

Participants were diagnosed with MCI using the international consensus criteria (Winblad et al., 2004). Specifically, the presence of cognitive impairment as determined by performance on a neuropsychological measure of at least 1.5 standard deviations below published normative values for age and/or education on a test battery covering five cognitive domains (memory, attention/information processing, language, spatial and executive abilities), a subjective complaint of decline in memory or other cognitive function either from the participant or informant, and normal or minimally impaired instrumental activities of daily living attributable to cognitive impairment (total average score <3.0 on the Bayer Activity of Daily Living Scale, Hindmarch et al., 1998).

MCI were classified into two subtypes (aMCI or naMCI) according to cognitive impairment profiles (Petersen, 2004). Participants with no impairments on neuropsychological tests were deemed to have normal cognition. In this study, we included individuals who had MRI scans from both baseline and 2-year follow-up (wave-2), and a wave-2 diagnosis of either cognitively normal or MCI. Demographic characteristics were detailed in Table 1. A total of 184 participants met these criteria, including 115 cognitively normal (CN), 42 aMCI, and 27 naMCI. The MRI measurements used in the present study have been previously published (Liu et al., 2013).

**Table 1:** Demographic characteristics of the sample.

**Time point**	**Diagnostic group**	**No. of subjects (male)**	**Age mean (*SD*)**	**Years of Edu mean (*SD*)**	**MMSE score mean *(SD)***
Baseline	Total	184 (91)	77.48 (4.40)	11.79 (3.60)	28.16 (1.32)
	CN	117 (56)	77.12 (4.43)	11.93 (3.53)	28.39 (1.23)
	aMCI	40 (28)	78.36 (4.11)	11.81 (3.96)	27.58 (1.32)
	naMCI	27 (7)	77.76 (4.65)	11.14 (3.42)	28.00 (1.44)
Wave-2	Total	184 (91)	79.38 (4.40)	11.79 (3.60)	28.40 (1.41)
	CN	115 (53)	78.78 (4.15)	12.06 (3.42)	28.83 (1.16)
	aMCI	42 (30)	81.26 (4.98)	11.87 (4.16)	27.64 (1.59)
	naMCI	27 (8)	79.03 (3.72)	10.49 (3.27)	27.78 (1.40)

*CN, cognitively normal; aMCI, amnestic mild cognitive impairment (MCI); naMCI, non-amnestic MCI; Edu, education, MMSE, Mini-mental state examination*.

### Image acquisition

MRI scans were obtained with a 3-T system (Philips Medical Systems, Best, The Netherlands) using the same sequence for both baseline and follow-up scans: *TR* = 6.39 ms, *TE* = 2.9 ms, flip angle = 8°, matrix size = 256 × 256, FOV = 256 × 256 × 190 mm, and slice thickness = 1 mm with no gap, yielding 1 × 1 × 1 mm^3^ isotropic voxels.

### Image processing

#### Sulcal measures

Cortical sulci were extracted from the images via the following steps. First, non-brain tissues were removed to produce images containing only GM, white matter (WM) and cerebrospinal fluid (CSF). This was done by warping a brain mask defined in the standard space back to the T1-weighted structural MRI scan. The brain mask was obtained with an automated skull stripping procedure based on the SPM5 skull-cleanup tool (Ashburner, 2009). Individual sulci were identified and extracted using the BrainVisa (BV, version 3.2) sulcal identification pipeline (Rivière et al., 2009). A sulcal labeling tool incorporating 500 artificial neural network-based pattern classifiers (Riviere et al., 2002; Sun et al., 2007) was used to label sulci. Sulci that were mislabeled by BV were manually corrected. For each hemisphere, we determined the average sulcal width for five sulci: superior frontal, intra-parietal, superior temporal, central, and the sylvian fissure. Sulcal width was defined as the average 3D distance between opposing gyral banks along the normal projections to the medial sulcal mesh (Kochunov et al., 2012). The five sulci investigated in the present study were chosen because they were present in all individuals, large and relatively easy to identify after facilitating error detection and correction, and located on different cerebral lobes. For each hemisphere, we calculated the global sulcal index (g-SI) as the ratio between the total sulcal area and outer cortical area (Penttilae et al., 2009). We calculated the g-SI of each brain with no manual intervention using BV.

#### Cortical thickness, GM volume

We computed average regional GM volume, average regional cortical thickness using the longitudinal stream in FreeSurfer 5.1 (http://surfer.nmr.mgh.harvard.edu/) (Reuter et al., 2012). This stream specifically creates an unbiased specific within-subject template space and image using robust, inverse consistent registration (Reuter and Fischl, 2011; Reuter et al., 2012). Briefly, this pipeline included the following processing steps, skull stripping, Talairach transforms, atlas registration, spherical surface maps, and parcellation of cerebral cortex (Desikan et al., 2006; Reuter et al., 2012). We applied Desikan parcellation (Desikan et al., 2006) which resulted 34 cortical regions of interest (ROIs) in each hemisphere. We visually inspected registration and segmentation. Scans were excluded if they failed visual quality control, resulting in an unequal number of scans available for different brain structures. We calculated both the cortical thickness and the regional volumes for every cortical regions of the Desikan parcellation.

#### Subcortical volume

Subcortical brain structures were extracted using FSL's FIRST (FMRIB Image Registration and Segmentation Tool, Version 1.2), a model-based segmentation/registration tool (Patenaude et al., 2011). We included the following left and right subcortical structures: thalamus, caudate, putamen, pallidum, hippocampus, amygdala, and nucleus accumbens. Briefly, the FIRST algorithm modeled each participant's subcortical structure as a surface mesh, using a Bayesian model incorporating a training set of all images. We conducted visual quality control of FSL results using ENIGMA protocols (http://enigma.ini.usc.edu/). Three slices of each of coronal, sagittal and axial planes were extracted from each linearly transformed brain. For comparison, an outline of the templates was mapped onto the slices. We confirmed that the size of the participant brain corresponded with that of the template, verified that the lobes were appropriately situated, and confirmed that the orientation of the participant matched the template.

#### WMHs

WMHs were delineated from coronal plane 3D T1-weighted and Fluid Attenuated Inversion Recovery (FLAIR) structural image scans using a pipeline described in detail previously (Wen et al., 2009). For each hemisphere, we calculated WMH volumes of eight brain regions: temporal, frontal, occipital, parietal, ventricle body, anterior horn, posterior horn, and cerebellum.

We obtained neuroimaging measurements of all participants at baseline and wave-2. The changes and the means values of those measurements were considered as the longitudinal features. There were altogether 178 MRI measurements for baseline and wave-2 feature sets, which included 12 sulcal measurements, 68 thickness measurements, 68 volume measurements, 14 subcortical measurements, and 16 WMH measurements. With the means and the changes, the longitudinal feature set included 356 MRI measurements.

### Feature selection

The aims of feature selection were to maximize the performance of classification by identifying the most discriminative features, and help in understanding the neuropathological basis of neurocognitive impairments such as MCI and dementia. Supervised feature selection methods were often divided into three categories, namely “filter,” “wrapper,” and “embedded,” respectively (Mwangi et al., 2014). A particular problem of those methods was that when they were applied in the neuroimaging fields, where the number of features largely exceeded the number of examples, the cross-validation based error estimates usually led to results with extremely large variances (Dougherty et al., 2010; Tohka et al., 2016). We proposed a feature selection method in this study to reduce the variances by integrating the filter and the wrapper procedures within the subsampling iterations. The optimal feature subset consisted of the features which were most frequently selected in all the subsamples of data. The discriminative abilities of the features were assessed in terms of the selection frequencies.

**Figure 1:**
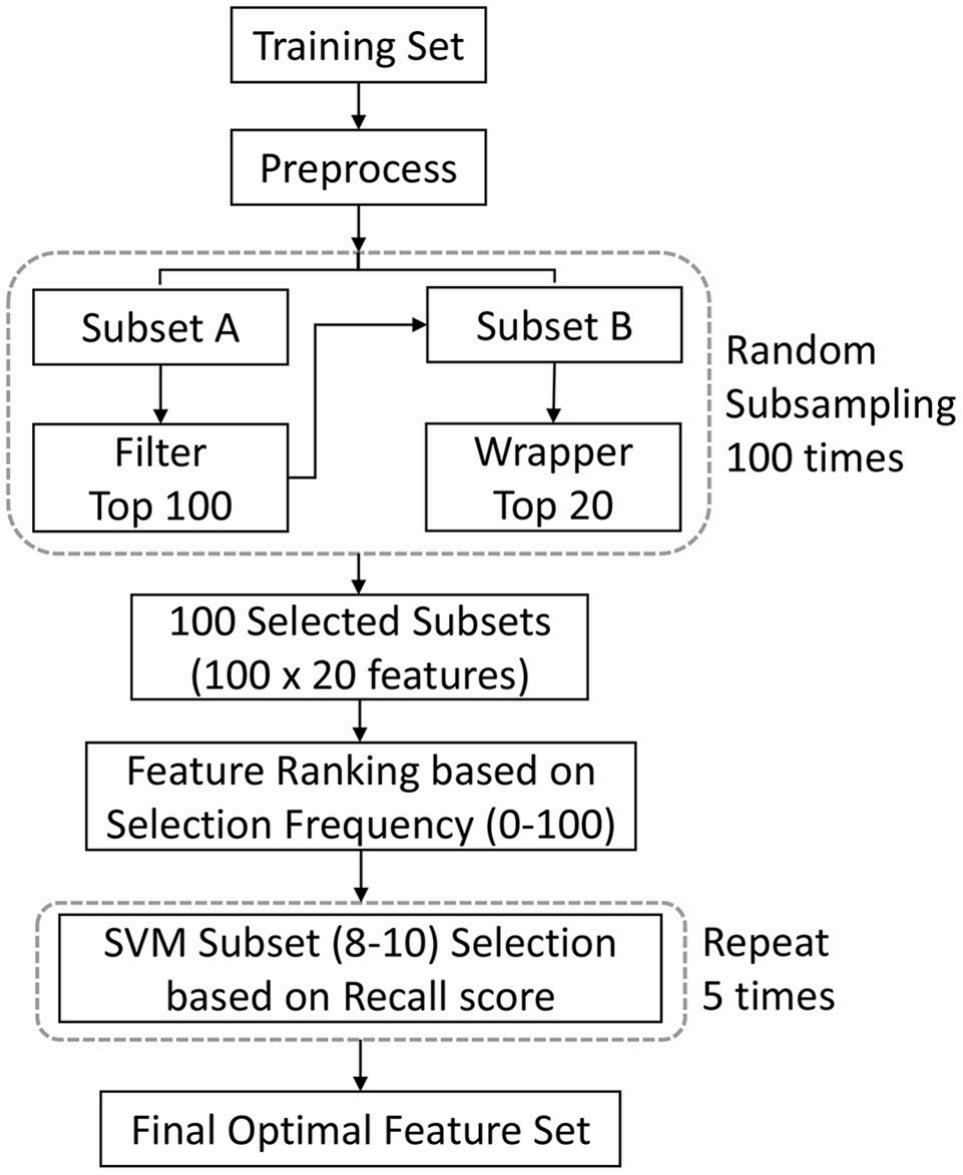
Illustration of the feature selection procedure. This procedure integrate filter and wrapper methods within the subsampling procedure. The optimal features consisted of the features which were most frequently selected in all the subsamples of data. The final optimal feature set was determined by validating classification performance on the training data. We used feature ranking with ANOVA F-value as the filtering process, and the recursive feature elimination algorithm as the wrapping process. A single experiment within a cross-validation (CV) iteration is depicted. SVM = support vector machine.

Figure 1 shows the flowchart of the feature selection procedure used in our study. We first randomly subsampled the training set 100 times. During each subsampling iteration, data were divided into two subsets of equal size, subset A and subset B. Subset A was processed by a filter to select features. The selected features were then applied to subset B. The subset B was processed by a wrapper to further reduce the number of features. After the subsampling processes, features were subsequently ranked in order of selection frequencies. The final optimal feature set was then determined by validating classification performance on the training data, using features chosen on the basis of frequency rank thresholds.

In the filter stage, ANOVA (analysis of variance) *F*-value were used to rank features on the basis of correlations with their diagnostic label. The top 100 features were selected at this stage. Then in the wrapping stage, the recursive feature elimination algorithm (Guyon et al., 2002) was used to further remove less informative features. Among the top 100 features, 20 were retained in this stage. The selection frequencies could be 100 at maximum or 0 at minimum. To mitigate the curse-of-dimensionality problem, the final feature set was limited with less than 10 features, and a variation section was established for the feature set to achieve the best validation performance. Given a frequency rank threshold Nf (Nf ϵ [10, 9, 8]), we randomly split the training data into 2 subgroups: one for training a SVM (Vapnik, 1995) classifier with top Nf features, and the other for validation. The kernel for the SVM is the radial basic function (rbf). This step was repeated 5 times, and the recall scores were computed (the recall score is the ratio Tp/(Tp + Fn), where Tp is the number of true positives and Fn is the number of false negatives). We chose the recall score as the criteria to minimize the impact of sample proportion imbalance. The top Nf features with the highest average recall score became the optimal feature set. We also evaluated the selected features using 2-tailed *t*-test.

### Classification and validation

**Figure 2:**
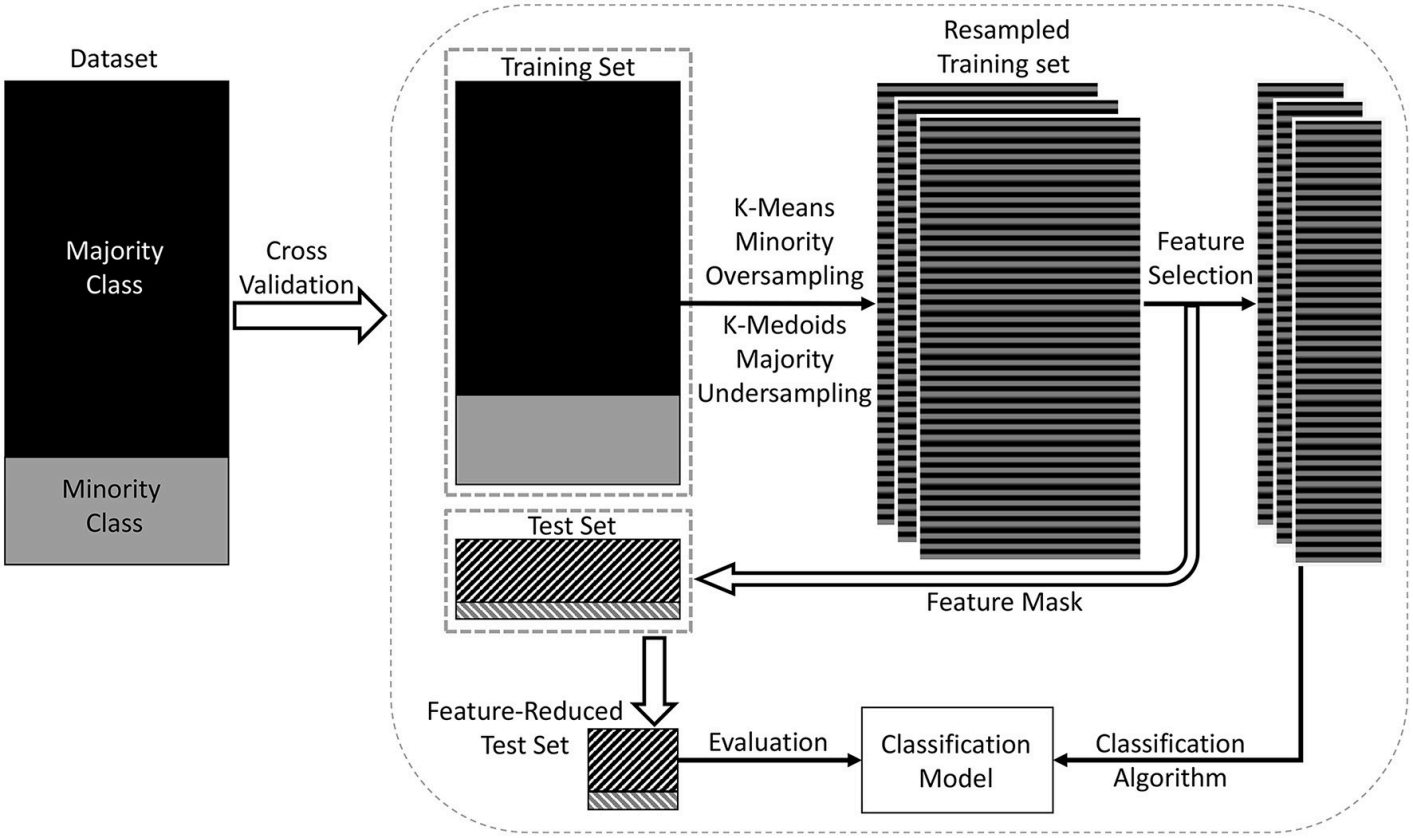
Overview of the proposed classification model. In this model, a training set and a test set were derived from the dataset using data points from both majority and minority classes (shown in the left rectangle of the figure). A combination of oversampling and undersampling technique was applied to the training set to generate a resampled training set. The training set in each cross-validation iteration was resampled three times to reduce the bias due to random dataset generation. Then feature selection was applied to select the most discriminative features. Then the classification model was trained on the dimension-reduced training set, and evaluated on the test set.

The imbalance of the sample could lead to a suboptimal classification performance. This study investigated a population-based sample, consisting of more cognitively normal individuals than MCI. There was also a large difference between the sample sizes of different MCI subtypes. We addressed this problem by using the data-resampling technique (Chawla et al., 2002; Dubey et al., 2014). An overview of the procedure is shown in Figure 2. We used a combination of oversampling and undersampling (Batista et al., 2004). K-means clustering (Macqueen, 1967) algorithm was used for oversampling, where new synthetic data were generated by clustering the minority class data. Briefly, Ns samples were clustered into Ns/3 clusters, and Ns/3 centroids were generated. Then these centroids and the original samples were combined for the next iteration of oversampling. The oversampling procedure was repeated until the size of minority class was 2/3 the size of the majority class. K-Medoids clustering (Hastie et al., 2001) algorithm was used for undersampling, where actual data points from the majority class were chosen as the cluster centers. The final training set was a combination of the oversampled minority class data and the undersampled majority class data. While resampling the training set, the test set remained the same. The training set was resampled 3 times to reduce the bias due to random data generation. Then the feature selection method was applied on those resampled training sets, thus producing 3 learning models. These models were combined using majority voting, where the final label of an instance was decided based on the majority votes received from all the models.

We chose Voting Classifier for classification (Maclin and Opitz, 1999). A Voting Classifier combines conceptually different machine learning classifiers and uses a majority vote or the average predicted probabilities (soft vote) to predict the class labels. The advantage of Voting Classifier is to balance out the individual weaknesses of a set of equally well performing models. We chose SVM (rbf kernel), Logistic Regression (LR) (Cox, 1958), and Random Forest (RF) (Breiman, 2001) as the estimators of the Voting Classifier. All the estimators were with default settings of parameters. Specific weights (1:4:1) were assigned to SVM, LR and RF via the weights parameter. The weights were selected experimentally to aim at a better sensitivity score. We started with the equal weights (1:1:1), and changed the weights to obtain the best results. The predicted class probabilities of each classifier were collected, multiplied by the weights of classifiers, and averaged. The final class label was then derived from the class label with the highest average probability. As different features had different scales, we standardized all the training data within a 0–1 range, and the same procedure was then applied to the test data.

We evaluated our method using stratified Shuffle Split cross-validation procedure, also known as Monte Carlo cross-validation (Berrar et al., 2007), which returned stratified randomized folds by preserving the percentage of samples for each class. The cross-validation procedure was repeated 10 times with a fixed 9:1 train-test ratio. The final classification results represented the average of these 10 independent experiments. We applied four metrics to assess the performance of the model: the accuracy, the specificity, the sensitivity, and the area under the receiver operating characteristic curve (AUC). AUC is a better measure than accuracy in imbalanced data sets and real-world applications (Huang and Ling, 2005; Bekkar et al., 2013).

It was important to note that we obtained a unique set of selected features in each training set. The training set in each cross-validation iteration was resampled 3 times, thus producing 3 resampled training sets. In each training set, the maximum possible selection frequency of one feature was 100. Considering the feature selection and data-resampling steps within the 10-iteration cross-validation procedure, the final maximum possible selection frequency of each feature was 3 × 100 × 10 = 3,000.

All the data processing and analyzing were performed using Python libraries Numpy 1.10.4 (Walt et al., 2011) and Scipy 0.17.0 (Jones et al., 2001) on Python 2.7.11 (Anaconda 4.0.0–64 bit, http://www.continuum.io/). All the machine learning methods were performed using the library Scikit-Learn 0.17.1 (Pedregosa et al., 2011).

## Results

### MCI subtypes classification

As shown in Table 2, in the classification of aMCI and CN, compared with using baseline features, using longitudinal features improved the performance to accuracy of 73%, sensitivity of 53%, specificity of 80%, and AUC of 0.75; the results of using longitudinal features were not superior to that using wave-2 features. Identifying naMCI from CN was relatively difficult considering the poor sensitivity value and AUC; the results of using longitudinal and cross-sectional features were comparable and without significant difference. In the classification of naMCI vs. aMCI, compared with using longitudinal features, using baseline features achieved better performance; the results of using wave-2 features were not significantly different from using longitudinal features.

**Table 2:** Classification results of MCI subtypes: features measured at baseline, wave-2 and longitudinally are used and compared.

**Task**	**No. of minority class**	**No. of majority class**	**Method**	**Accuracy (%)**	**Sensitivity (%)**	**Specificity (%)**	**AUC**
aMCI vs. CN	aMCI = 42	CN = 115	Baseline	0.64	0.42	0.71	0.68
			Wave-2	0.81^*^	0.68	0.85	0.74
			Longitudinal	0.73	0.53	0.80	0.75
naMCI vs. CN	naMCI = 27	CN = 115	Baseline	0.67	0.37	0.75	0.57
			Wave-2	0.65	0.30	0.74	0.58
			Longitudinal	0.70	0.23	0.82	0.60
naMCI vs. aMCI	naMCI = 27	aMCI = 42	Baseline	0.77^*^	0.70^*^	0.82	0.84^*^
			Wave-2	0.71	0.57	0.82	0.70
			Longitudinal	0.61	0.40	0.78	0.71

*wave-2, 2-year follow-up; MCI, mild cognitive impairment; CN cognitively normal; aMCI, amnestic MCI; naMCI, non-amnestic MCI; AUC, area under the receiver operating characteristic curve*.

* *Significantly different from the method using longitudinal features; results are from t-test (p < 0.05)*.

### Discriminative features

The discriminative ability of the features used in this study were assessed by examining the frequency with which they were selected. We listed the top 10 most frequently selected features in each MCI subtype classification experiment (see Tables 3–5). In the comparison of aMCI vs. CN, thickness of right frontal pole, left superior temporal, volume of right thalamus, and right hippocampus were more discriminative than the rest of features (see Table 3). In the classification of naMCI vs. aMCI, thickness of right rostral middle frontal, right pericalcarine, right frontal pole, and volume of right rostral anterior cingulate were more discriminative than the others (see Table 5). Regardless of cross-sectional (baseline and wave-2) or longitudinal, all the features mentioned above were listed in the top-10 feature list. In the naMCI vs. CN comparison, volume of left temporal pole and right amygdala were also discriminative (see Table 4).

**Table 3:** Selected features for the classification of aMCI vs. CN.

**Task**	**Baseline feature**	**Frequency**	**p^a^**	**Wave-2 feature**	**Frequency**	**p^a^**	**Longitudinal feature**	**Frequency**	**p^a^**
***aMCI vs. CN***	***Right frontal pole thickness***	***2,658***	***0.001***	***Right frontal pole thickness***	***2,786***	*<**0.001***	***Right frontal pole thickness***	***2,715***	*<**0.001***
	***Right thalamus volume***	***2,136***	***0.006***	***Right hippocampus volume***	***2,105***	*<**0.001***	***Right thalamus volume***	***2,470***	***0.001***
	***Left superior temporal thickness***	***1,620***	***0.001***	***Right thalamus volume***	***1,775***	***0.001***	***Right hippocampus volume***	***2,071***	***0.001***
	Right hippocampus volume	1,344	0.005	Left superior temporal thickness	1,144	0.005	Left superior temporal thickness	1,212	0.002
	Right g-SI^c^	1,265	0.003	Left sucal width of superior frontal	1,062	0.002	Right sucal width of superior temporal^b^^*^	1,036	0.027
	Right transverse temporal thickness^c^	755	0.013	Right sucal width of superior frontal^c^	963	0.001	Right pericalcarine thickness	876	0.004
	Right pericalcarine thickness	736	0.005	Right amygdala volume^c^	963	0.073	Left precentral thickness^b^^*^	869	0.035
	Right rostral anterior cingulate volume^c^	693	0.128	Right pericalcarine thickness	958	0.006	Left inferior temporal thickness^*^	827	0.019
	Right paracentral thickness^c^	637	0.022	Right accumbens volume^c^	660	0.011	Right paracentral thickness^b^^*^	819	0.012
	Left posterior cingulate volume^c^	545	0.134	Left medial orbitofrontal thickness^c^	633	0.045	Right sulcal width of superior frontal	722	0.002

*A feature measured at baseline, wave-2 or longitudinally is defined as baseline feature, wave-2 feature or longitudinal feature, respectively. The first 10 most frequently selected features and their selection frequencies are listed. The maximum possible selection frequency of each feature is 3000. The features with selection frequencies above 1500 are in bold. wave-2, 2-year follow-up; MCI, mild cognitive impairment; CN, cognitively normal; aMCI, amnestic MCI*.

a *Results for comparisons of positive subjects and negative subjects using t-tests*.

b *Changes measurements, the rest longitudinal features are means measurements*.

c *Features that were selected at a single time point (either at baseline or wave-2)*.

* *Features that were selected only in longitudinal case*.

**Table 4:** Selected features for the classification of naMCI vs. CN.

**Task**	**Baseline feature**	**Frequency**	**p^a^**	**Wave-2 feature**	**Frequency**	**p^a^**	**Longitudinal feature**	**Frequency**	**p^a^**
***naMCI vs. CN***	***Right WMH volume of cerebellum**^c^*	***2,240***	***0.014***	***Left lateral occipital thickness**^c^*	***2,087***	***0.002***	***Right entorhinal volume***^b^^*^	***2,866***	*<**0.001***
	***Left temporal pole volume***	***2,227***	***0.072***	***Right rostral middle frontal thickness***	***1,670***	***0.024***	***Right amygdala volume***	***1,852***	***0.001***
	***Right amygdala volume***	***2,027***	***0.002***	***Left temporal pole volume***	***1,636***	***0.086***	***Right posterior cingulate volume***^b^^*^	***1,608***	***0.008***
	***Right rostral middle frontal thickness***	***1,757***	***0.008***	***Right amygdala volume***	***1,527***	***0.003***	Left lateral occipital thickness	1,434	0.002
	***Right rostral anterior cingulate volume***	***1,718***	***0.011***	Right sucal width of superior frontal ^c^	1,259	0.017	Left temporal pole volume	1,256	0.074
	Left middle temporal thickness ^c^	1,316	0.002	Left pericalcarine volume ^c^	1,218	0.012	Left posterior cingulate thickness^b^^*^	891	0.022
	Right inferior parietal thickness ^c^	953	0.002	Right rostral anterior cingulate volume	993	0.026	Left amygadala volume^b^^*^	746	0.117
	Right thalamus volume ^c^	833	0.005	Right putamen volume ^c^	754	0.001	Left temporal pole thickness^b^^*^	630	0.054
	left transverse temporal volume ^c^	778	0.182	Right supramarginal volume	602	0.263	Left middle temporal thickness	613	0.007
	Right supramarginal volume	638	0.108	Left sulcal width of superior temporal ^c^	515	0.134	Right WMH volume of cerebellum	578	0.378

*A feature measured at baseline, wave-2 or longitudinally is defined as baseline feature, wave-2 feature or longitudinal feature, respectively. The first 10 most frequently selected features and their selection frequencies are listed. The maximum possible selection frequency of each feature is 3000. The features with selection frequencies above 1,500 are in bold. wave-2, 2-year follow-up; CN, cognitively normal; naMCI, non-amnestic MCI*.

a *Results for comparisons of positive subjects and negative subjects using t-tests*.

b *Changes measurements, the rest longitudinal features are means measurements*.

c *Features that were selected at a single time point (either at baseline or wave-2)*.

* *Features that were selected only in longitudinal case*.

**Table 5:** Selected features for the classification of naMCI vs. aMCI.

**Task**	**Baseline feature**	**Frequency**	**p^a^**	**Wave-2 feature**	**Frequency**	**p^a^**	**Longitudinal feature**	**Frequency**	**p^a^**
***naMCI vs. aMCI***	***Right rostral middle frontal thickness***	***2,643***	*<**0.001***	***Right rostral anterior cingulate volume***	***2,754***	*<**0.001***	***Right rostral anterior cingulate volume***	***2,598***	*<**0.001***
	***Right rostral anterior cingulate volume***	***2,538***	*<**0.001***	***Right frontal pole thickness***	***2,634***	*<**0.001***	***Right rostral middle frontal thickness***	***2,502***	*<**0.001***
	***Right pericalcarine thickness***	***2,241***	***0.001***	***Right rostral middle frontal thickness***	***2,190***	*<**0.001***	***Right frontal pole thickness***	***2,478***	*<**0.001***
	***Right frontal pole thickness***	***1,815***	*<**0.001***	***Right pericalcarine thickness***	***1,551***	***0.005***	***Right pericalcarine thickness***	***1,830***	***0.002***
	***Right g-si***^c^	***1,539***	***0.004***	Left transverse temporal volume^c^	1,131	0.028	Right lateral occipital thickness	1,062	0.001
	Right lateral occipital thickness^c^	1,023	< 0.001	Right wmh volume of frontal^c^	1,071	0.122	Right entorhinal volume^b^^*^	1,029	0.010
	Right transverse temporal thickness^c^	750	0.014	Left rostral middle frontal volume^c^	813	0.037	Left transverse temporal thickness	867	0.009
	Left inferior temporal thickness^c^	687	< 0.001	Right insula thickness^c^	678	0.037	Left inferior temporal thickness	666	0.001
	Right parsorbitalis thickness^c^	666	0.002	Right frontal pole volume^c^	573	0.054	Left precentral thickness^*^	639	0.001
	Right transverse temporal thickness^c^	480	0.011	Right sulcal width of superior temporal^c^	552	0.026	Right g-SI	591	0.011

*A feature measured at baseline, wave-2 or longitudinally is defined as baseline feature, wave-2 feature or longitudinal feature, respectively*.

*The first 10 most frequently selected features and their selection frequencies are listed. The maximum possible selection frequency of each feature is 3,000. The features with selection frequencies above 1500 are in bold. Key: wave-2, 2-year follow-up; aMCI, amnestic MCI; naMCI, non-amnestic MCI*.

a *Results for comparisons of positive subjects and negative subjects using t-tests*.

b *Change measurement, the rest longitudinal features are mean measurements*.

c *Features that were selected at a single time point (either at baseline or wave-2)*.

* *Features that were selected only in longitudinal case*.

**Figure 3:**
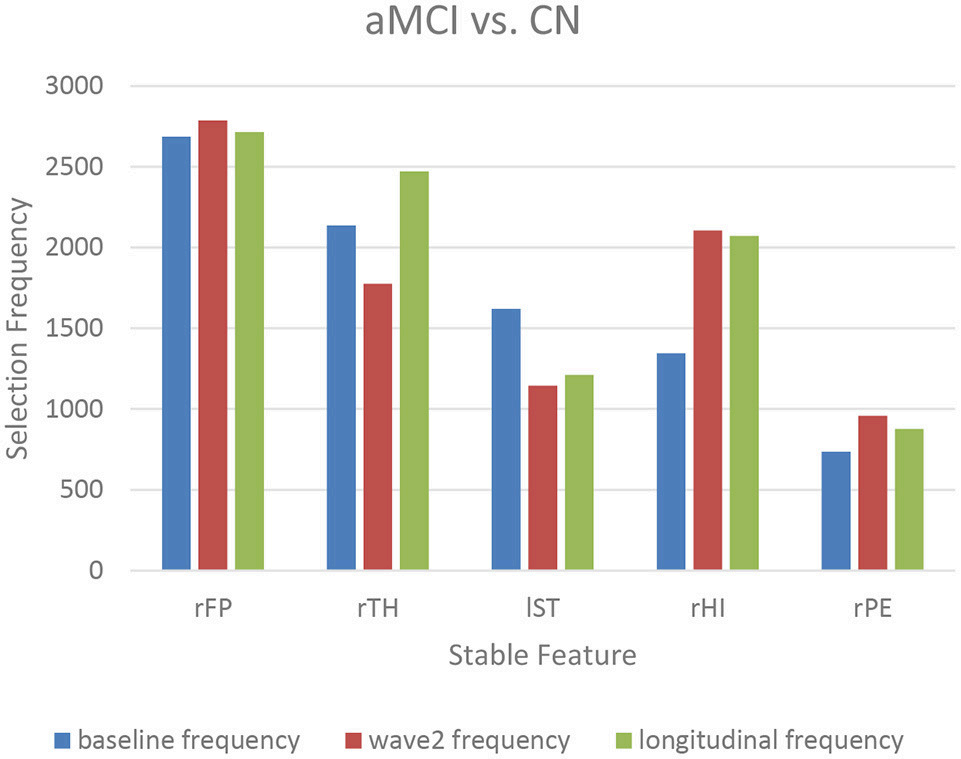
The selection frequencies of the stable features for aMCI vs. CN classification. The baseline, wave-2 or longitudinal frequency are the selection frequencies of the feature measured at baseline, wave-2 or longitudinally, respectively. The selection frequency (between 0 and 3,000) of each feature is indicative of the discriminative power for classification. Thickness of right frontal pole is stable across time. Volume of right thalamus and left superior temporal provides more information in former time point, while the volume of right hippocampus is more discriminative in later time point. rFP, right frontal pole thickness; rTH, right thalamus volume; lST, left superior temporal thickness; rHI, right hippocampus volume; rPE, right pericalcarine thickness.

**Figure 4:**
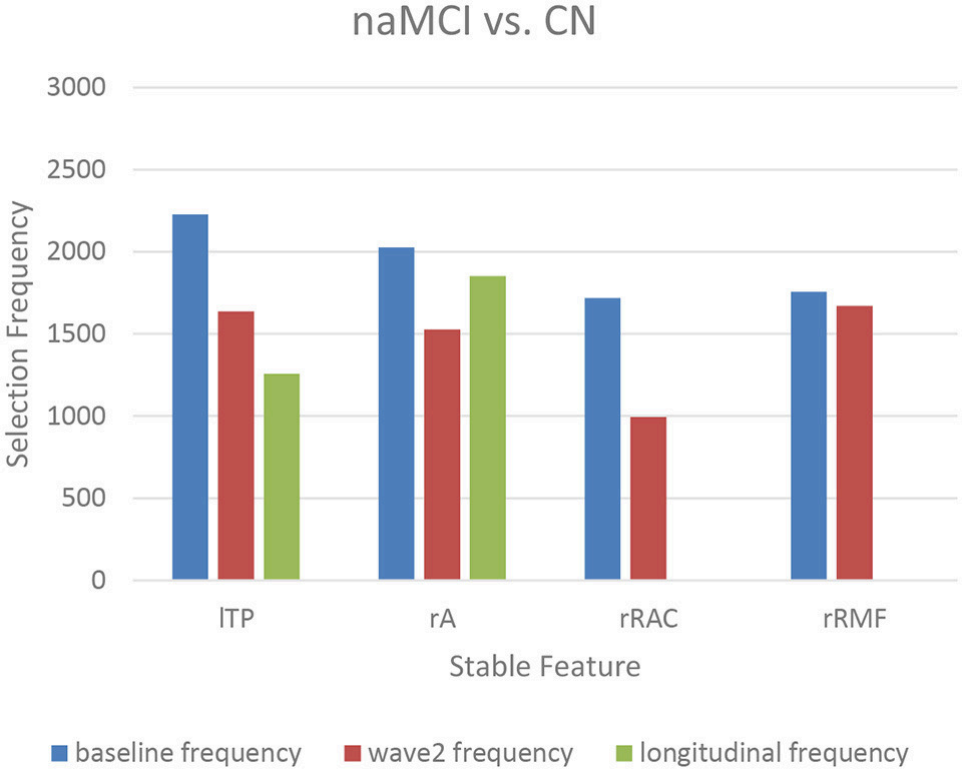
The selection frequencies of the stable features for naMCI vs. CN classification. The baseline, wave-2 or longitudinal frequency are the selection frequencies of the feature measured at baseline, wave-2 or longitudinally, respectively. The selection frequency (between 0 and 3,000) of each feature is indicative of the discriminative power for classification. Volume of left temporal pole is a more important biomarker in former time point. When measured longitudinally, volume of right rostral anterior cingulate and thickness of right middle frontal are not selected in the first 10 feature list. The right amygdala volume is stable over time. lTP, left temporal pole volume; rA, right amygdala volume; rRAC, right rostral anterior cingulate volume; rRMF, right rostral middle frontal thickness.

**Figure 5:**
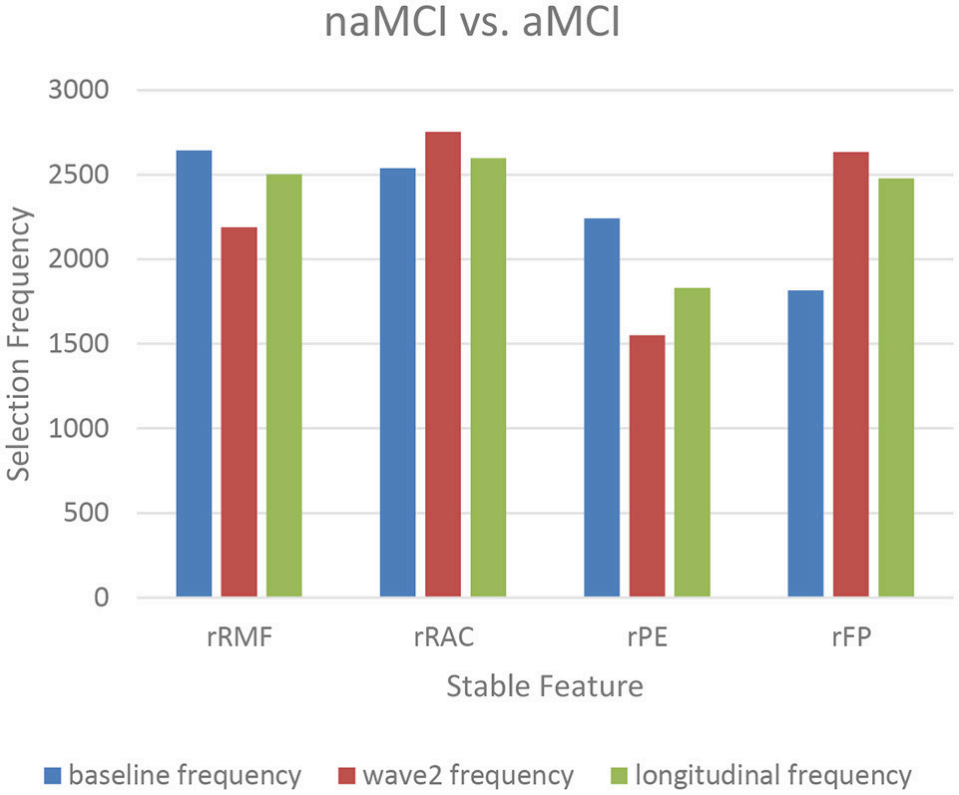
The selection frequencies of the stable features for naMCI vs. aMCI classification. The baseline, wave-2 or longitudinal frequency are the selection frequencies of the feature measured at baseline, wave-2 or longitudinally, respectively. The selection frequency (between 0 and 3,000) of each feature is indicative of the discriminative power for classification. Volume of right rostral middle frontal and thickness of right pericalcarine are more discriminative in former time point, while volume of right frontal pole is more discriminative in later time point. And volume of right rostral anterior cingulate provide important information at all-time points. rRMF, right rostral middle frontal thickness; rRAC, right rostral anterior cingulate volume; rPE, right pericalcarine thickness; rFP, right frontal pole volume.

The top-10 selected features were analyzed to identify the temporal patterns. Several features measured at different time points showed dynamic discriminative powers. Figures 3–5 shows the selection frequencies of the stable features measured at each time point. A feature may be identified as stable when this feature was selected at all the baseline, wave-2, and longitudinally. The selection frequencies of the stable features for aMCI vs. CN classification are shown in Figure 3. We observed that thickness of right frontal pole was a stable biomarker, since its selection frequencies were close between different time points. The selection frequencies of several biomarkers changed visibly over time, including volume of right thalamus, right hippocampus, and thickness of left superior temporal. In the classification of naMCI vs. CN (see Figure 4), only a few features were stable. We observed that the volume of right amygdala provided more useful information at baseline. Volume of left temporal pole and right rostral cingulate carried more information at baseline. In the classification of naMCI vs. aMCI (see Figure 5), volume of right rostral middle frontal and thickness of right pericalcarine thickness were selected more often at baseline, while volume of right frontal pole were more discriminative at wave-2. And volume of right rostral anterior cingulate provided important information at all-time points.

Furthermore, some features were selected in the top-10 feature list at either baseline or wave-2, such as the right g-SI index, sucal width of superior frontal (see Table 3); thickness of left lateral occipital, WMH volume of right cerebellum (see Table 4); thickness of right lateral occipital, and WMH volume of right frontal (see Table 5). On the other hand, some features were selected only in longitudinal cases, such as sulcal width of right superior temporal, thickness of left inferior temporal (see Table 3); volume of right entorhinal and right posterior cingulate, thickness of left posterior cingulate and temporal pole (see Table 4); thickness of left precentral, volume of right entrohinal (see Table 5). Most of these longitudinal features were the differences (changes value) between the measures of two time points.

## Discussion

Our study examined classification of MCI subtypes in community-dwelling elderly using cross-sectional and longitudinal MRI measurements. Our classification framework implemented a data-resampling step to reduce the effect of the class-imbalance, and a feature selection step in which maximally most discriminative feature subsets were identified. The results suggested that individuals with aMCI could be differentiated from CN and naMCI with MRI-based biomarkers, but identifying naMCI from CN was still a challenge. Identifying aMCI from CN using longitudinal features achieved better performance than that using baseline features, but the results were not superior to that using wave-2 features. The best performance of differentiating aMCI from naMCI was achieved with baseline features. In addition, we analyzed and identified the dynamics of the biomarkers.

The subtlety of brain changes in MCI challenges the image-based classification. Previous studies reported using machine learning to differentiate MCI from cognitively normal (Wee et al., 2011, 2012; Zhang et al., 2011, 2017; Cui et al., 2012b; Liu et al., 2015, 2017). Cui et al. used combined measurements of T1-weighted and diffusion tensor imaging (DTI) to distinguish aMCI from CN, achieved a classification accuracy of 71%, sensitivity 52%, specificity 78%, and AUC 0.70 (Cui et al., 2012b). Our performance (accuracy 81%, sensitivity 68%, specificity 85%, and AUC 0.74) is better than their study. The approach of Wee et al. was a kernel combination method that utilized DTI and resting-state functional magnetic resonance imaging (Wee et al., 2012). Although their classification accuracy of 96.3% is higher than ours, the inclusion of multi-modality imaging could restrict their use in clinical settings, and the small sample size of fewer than 30 participants may also make their results less robust. Considering the heterogeneity of MCI, we performed MCI subtypes classification, and the results demonstrated that aMCI and naMCI could be accurately separated with MRI biomarkers. And the results showed that the various groups demonstrated different patterns of atrophy on MRI. However, differentiating naMCI from CN was difficult considering the low sensitivities (see Table 2). The serious imbalance of classes could result in this poor performance, although we had performed data-resampling to mitigate the difference of the sample sizes. Compared with aMCI, naMCI individuals are more likely to revert to normal cognition (Roberts et al., 2014; Aerts et al., 2017). The MCI individuals who reverted might have different underlying mechanisms (Zhang et al., 2012b). In addition, higher estimates of MCI incidence in clinic-based studies (Petersen, 2004, 2010) than in population-based studies suggested that the rate of reversion to normal cognition may be lower in the clinic setting than in population-based studies (Koepsell and Monsell, 2011; Lopez et al., 2012) such as ours.

Longitudinal patterns of atrophy identified in MRI measurements can be used to elevate the prediction of cognitive decline (Rusinek et al., 2003; Risacher et al., 2010). McEvoy et al. investigated whether single-time-point and longitudinal volumetric MRI measures provided predictive prognostic information in patients with aMCI. Their results showed that the information regarding the rate of atrophy progression over a 1-year period improved risk prediction compared with using single-time-point MRI measurement (McEvoy et al., 2011). Huang et al. used longitudinal changes over 4 years of T1-weighted MRI scans to predict AD conversion in MCI subjects. Their results showed that the model with longitudinal data consistently outperformed the model with baseline data, especially achieved 17% higher sensitivity than the model with baseline data (Huang et al., 2017). In our study, the results showed that the longitudinal features failed to provide additional information for identifying aMCI and naMCI compared with cross-sectional features. In the classification of aMCI vs. CN, the accuracy with longitudinal features was nearly 10% higher than the accuracy with baseline features, but was not superior to the accuracy with wave-2 features (Table 2). The performance of using longitudinal features was comparable to using cross-sectional features at baseline and wave-2 for distinguishing naMCI from CN. In addition, the highest performance of distinguishing naMCI from aMCI was achieved with baseline features (see Table 2). This might because the progression of naMCI showed no coherent pattern of atrophy. The patterns of atrophy differ among aMCI and naMCI, and subjects with naMCI showed scattered patterns of gray matter loss without any particular focus (Whitwell et al., 2007). All the subjects of our study were community-dwelling. It was likely that the naMCI subjects had atrophy patterns closer to those of CN at baseline, but over the time the patterns progressed to more MCI-like at wave-2. Our results also indicated that features selected for identifying naMCI were unstable over time, which might be because clinical classification of naMCI can be based on impairment individually or in combination across a range of non-amnestic cognitive domains (language, visuo-spatial, processing speed, or executive abilities).

Longitudinal research has observed the dynamics of biomarkers (Trojanowski et al., 2010; Sabuncu et al., 2011; Eskildsen et al., 2013; Zhou et al., 2013). Some features provided significant information at all-time points while some other features were shown to be useful at a specific time point. Eskildsen et al. demonstrated that prediction accuracies of conversion from MCI to AD can be improved by learning the atrophy patterns that were specific to the different stages of disease progression (Eskildsen et al., 2013). They found that medial temporal lobe structures were stable biomarkers across all stages. Hippocampus was not discriminative at 36 months prior to AD diagnosis, but was included in all prediction cases of later stages. In addition, biomarkers were mostly selected from the cingulate gyrus, which is well known to be affected in early AD (Eskildsen et al., 2013). Histological studies suggest that the integrity of entorhinal cortex is among the first affected, which is then only later followed by an atrophy of the hippocampus (Braak et al., 1993). In our study, we also found that volume of the right hippocampus was more discriminative at wave-2 (see Figure 3, Table 3), which would complemented the histological findings. Furthermore, the thalamic volume was discriminative and stable over time (see Figure 3, Table 3), which was consistent with a previous study that the structure and function of thalamus determined severity of cognitive impairment (Schoonheim et al., 2015). Volume of left posterior cingulate and right rostral anterior cingulate were more discriminative at baseline for identifying aMCI and naMCI from CN (see Tables 3, 4), while volume of right rostral anterior cingulate was a stable biomarker for naMCI vs. aMCI classification over time (see Figure 5, Table 5). Zhou et al. used the baseline MRI features to predict MMSE (The Mini–Mental State Examination, Folstein et al., 1975) and ADAS-Cog (Alzheimer's Disease Assessment Scale cognitive subscale, Rosen et al., 1984) scores in the next 4 years (Zhou et al., 2013). They observed that the average cortical thickness of left middle temporal, left and right entorhinal, and volume of left hippocampus were important biomarkers for predicting ADAS-Cog scores at all-time points. Cortical volume of left entorhinal provided significant information in later stages than in the first 6 months. Several biomarkers including volume of left and right amygdala provided useful information only at later time points (Zhou et al., 2013). In our study, cross-sectional (both baseline and wave-2) volume of right entorhinal was not an important biomarker for the classification of naMCI vs. CN, but the longitudinal volume change of right entorhinal (see Table 4) was discriminative. Volume of right amygdala was discriminative at all-time points for naMCI vs. CN classification (see Figure 4, Table 4). The dynamics of biomarker could potentially aid in developing stable imaging biomarkers and in tracking the progression of cognitive impairment.

The use of same dataset for feature selection and classification is termed “double-dipping,” which will lead to distorted descriptive statistics and artificially inflated accuracies (Kriegeskorte et al., 2009; Pereira et al., 2009; Eskildsen et al., 2013; Mwangi et al., 2014). Due to the limited samples in neuroimaging studies, carelessly designed training, testing and validation schemes, the risk of double-dipping is high. Eskildsen et al. used cortical regions potentially discriminative for predicting AD. They found that by inclusion of test subjects in the feature selection process, the prediction accuracies were artificially inflated (Eskildsen et al., 2013). In our experiments, training datasets and test datasets were adequately separated using cross-validation procedure. The training set in each cross-validation iteration were used for data-resampling, feature selection and classifier training, while the test set were only used for validating classification performance.

The main limitation of the present study was the limited sample size. Our method required longitudinal data, thus limiting the subjects with MRI scans at both time points. Secondly, this study investigated a population-based sample, consisting of more cognitively normal individuals than MCI. There was also a difference between the sample sizes of aMCI and naMCI. The findings need to be replicated in other data sets.

## Conclusion

In conclusion, the present study investigated MCI subtypes classification in a sample from community-dwelling elderly using both cross-sectional and longitudinal MRI features. Our experiments suggested that longitudinal features were not superior to the cross-sectional features for MCI subtypes classifications. Dynamics of the biomarkers were analyzed and identified. Future studies with longer follow-up and more measurement occasions may lead to the better understanding of the trajectories for cognitive impairment.

## Author contributions

HG, TL, and JC: Study design, data analyses, interpretation of the results, manuscript writing. WW and DT: Study design, interpretation of the results. NK, PS, and HB: Data collection, interpretation of the results. JJ, JZ, HN, WZ, and YW: Data analyses. All authors participated in manuscript revision and final approval.

### Conflict of interest statement

The authors declare that the research was conducted in the absence of any commercial or financial relationships that could be construed as a potential conflict of interest.

## References

[B1] Aerts L. Heffernan M. Kochan N. A. Crawford J. D. Draper B. Trollor J. N.. (2017). Effects of MCI subtype and reversion on progression to dementia in a community sample. Neurology 88, 2225–2232. 10.1212/WNL.0000000000004015 28490651

[B2] Ardekani B. A. Bermudez E. Mubeen A. M. Bachman A. H. Alzheimer's Disease Neuroimaging Initiative. (2017). Prediction of incipient alzheimer's disease dementia in patients with mild cognitive impairment. J. Alzheimers. Dis. 55, 269–281. 10.3233/JAD-160594 27662309

[B3] Ashburner J. (2009). Computational anatomy with the SPM software. Magn. Reson. Imaging 27, 1163–1174. 10.1016/j.mri.2009.01.006 19249168

[B4] Batista G. E. A. P. A. Prati R. C. Monard M. C. (2004). A study of the behavior of several methods for balancing machine learning training data. SIGKDD Explor. Newsl. 6, 20–29. 10.1145/1007730.1007735

[B5] Bekkar M. Djemaa H. K. Alitouche T. A. (2013). Evaluation measures for models assessment over imbalanced data sets. J. Inform. Eng. Appli. 3, 27–38.

[B6] Berrar D. Granzow M. Dubitzky W. (2007). Introduction to genomic and proteomic data analysis, in Fundamentals of Data Mining in Genomics and Proteomics (Boston, MA: Springer).

[B7] Braak H. Braak E. Bohl J. (1993). Staging of Alzheimer-related cortical destruction. Eur. Neurol. 33, 403–408. 10.1159/000116984 8307060

[B8] Breiman L. (2001). Machine learning, volume 45, Number 1 - SpringerLink. Mach. Learn. 45, 5–32. 10.1023/A:1010933404324

[B9] Brodaty H. Heffernan M. Kochan N. A. Draper B. Trollor J. N. Reppermund S.. (2013). Mild cognitive impairment in a community sample: the sydney memory and ageing study. Alzheimers Dement. 9, 310–317.e311. 10.1016/j.jalz.2011.11.010 23110866

[B10] Bron E. E. Smits M. van der Flier W. M. Vrenken H. Barkhof F. Scheltens P.. (2015). Standardized evaluation of algorithms for computer-aided diagnosis of dementia based on structural MRI: the CADDementia challenge. Neuroimage 111, 562–579. 10.1016/j.neuroimage.2015.01.048 25652394 PMC4943029

[B11] Cai K. P. Xu H. Guan H. Zhu W. L. Jiang J. Y. Cui Y.. (2017). Identification of early-stage Alzheimer's disease using sulcal morphology and other common neuroimaging indices. PLoS ONE 12:e0170875. 10.1371/journal.pone.0170875 28129351 PMC5271367

[B12] Chawla N. V. Bowyer K. W. Hall L. O. Kegelmeyer W. P. (2002). SMOTE: synthetic minority over-sampling technique. J. Artif. Intell. Res. 16, 321–357. 10.1613/jair.953

[B13] Cox D. R. (1958). The regression analysis of binary sequences. J. R. Stat. Soc. 20, 215–242.

[B14] Cui Y. Sachdev P. S. Lipnicki D. M. Jin J. S. Luo S. Zhu W.. (2012a). Predicting the development of mild cognitive impairment: a new use of pattern recognition. Neuroimage 60, 894–901. 10.1016/j.neuroimage.2012.01.084 22289804

[B15] Cui Y. Wen W. Lipnicki D. M. Beg M. F. Jin J. S. Luo S.. (2012b). Automated detection of amnestic mild cognitive impairment in community-dwelling elderly adults: a combined spatial atrophy and white matter alteration approach. Neuroimage 59, 1209–1217. 10.1016/j.neuroimage.2011.08.013 21864688

[B16] Cuingnet R. Gerardin E. Tessieras J. Auzias G. Lehericy S. Habert M. O.. (2011). Automatic classification of patients with Alzheimer's disease from structural MRI: a comparison of ten methods using the ADNI database. Neuroimage 56, 766–781. 10.1016/j.neuroimage.2010.06.013 20542124

[B17] Davatzikos C. Bhatt P. Shaw L. M. Batmanghelich K. N. Trojanowski J. Q. (2011). Prediction of MCI to AD conversion, via MRI, CSF biomarkers, and pattern classification. Neurobiol. Aging 32, 2322.e19-27. 10.1016/j.neurobiolaging.2010.05.023 20594615 PMC2951483

[B18] Desikan R. S. Segonne F. Fischl B. Quinn B. T. Dickerson B. C. Blacker D.. (2006). An automated labeling system for subdividing the human cerebral cortex on MRI scans into gyral based regions of interest. Neuroimage 31, 968–980. 10.1016/j.neuroimage.2006.01.021 16530430

[B19] Dougherty E. R. Sima C. Hua J. P. Hanczar B. Braga-Neto U. M. (2010). Performance of error estimators for classification. Curr. Bioinform. 5, 53–67. 10.2174/157489310790596385

[B20] Dubey R. Zhou J. Y. Wang Y. L. Thompson P. M. Ye J. P. Alzheimer's Disease Neuroimaging Initiative. (2014). Analysis of sampling techniques for imbalanced data: an n = 648 ADNI study. Neuroimage 87, 220–241. 10.1016/j.neuroimage.2013.10.005 24176869 PMC3946903

[B21] Eskildsen S. F. Coupe P. Garcia-Lorenzo D. Fonov V. Pruessner J. C. Collins D. L.. (2013). Prediction of Alzheimer's disease in subjects with mild cognitive impairment from the ADNI cohort using patterns of cortical thinning. Neuroimage 65, 511–521. 10.1016/j.neuroimage.2012.09.058 23036450 PMC4237400

[B22] Falahati F. Westman E. Simmons A. (2014). Multivariate Data Analysis and Machine Learning in Alzheimer's Disease with a Focus on Structural Magnetic Resonance Imaging. J. Alzheimers Dis. 41, 685–708. 10.3233/JAD-131928 24718104

[B23] Folstein M. Folstein S. McHugh P. (1975). “Mini-mental state”: a practical method for grading the cognitive state of patients for the clinician. J. Psychiatr. Res. 12, 189–198. 10.1016/0022-3956(75)90026-6 1202204

[B24] Grundman M. Petersen R. C. Ferris S. H. Thomas R. G. Aisen P. S. Bennett D. A.. (2004). Mild cognitive impairment can be distinguished from Alzheimer disease and normal aging for clinical trials. Arch. Neurol. 61, 59–66. 10.1001/archneur.61.1.59 14732621

[B25] Guo S. Lai C. Wu C. Cen G. Alzheimer's Disease Neuroimaging I. (2017). Conversion discriminative analysis on mild cognitive impairment using multiple cortical features from MR Images. Front. Aging Neurosci. 9:146 10.3389/fnagi.2017.00146 28572766 PMC5435825

[B26] Guyon I. Weston J. Barnhill S. Vapnik V. (2002). Gene selection for cancer classification using support vector machines. Mach. Learn. 46, 389–422. 10.1023/A:1012487302797

[B27] Hastie T. Tibshirani R. Friedman J. (2001). The Elements of Statistical Learning. New York, NY: Springer series in statistics.

[B28] Hindmarch I. Lehfeld H. De J. P. Erzigkeit H. (1998). The bayer activities of daily living scale (B-ADL). Dement. Geriatr. Cogn. Disord. 9 (Suppl. 2), 20–26. 10.1159/000051195 9718231

[B29] Huang J. Ling C. X. (2005). Using, A. U. C., and accuracy in evaluating learning algorithms. IEEE Trans. Knowl. Data Eng. 17, 299–310. 10.1109/TKDE.2005.50

[B30] Huang M. Yang W. Feng Q. Chen W. Alzheimer's Disease Neuroimaging Initiative. (2017). Longitudinal measurement and hierarchical classification framework for the prediction of Alzheimer's disease. Sci. Rep. 7:39880. 10.1038/srep39880 28079104 PMC5227696

[B31] Jones E. Oliphant E. Peterson P. (2001). SciPy: Open Source Scientific Tools for Python. http://www.scipy.org/ (Accessed Jan 11, 2017)

[B32] Jung N.-Y. Seo S. W. Yoo H. Yang J.-J. Park S. Kim Y. J.. (2016). Classifying anatomical subtypes of subjective memory impairment. Neurobiol. Aging 48, 53–60. 10.1016/j.neurobiolaging.2016.08.010 27639121

[B33] Kochunov P. Rogers W. Mangin J. F. Lancaster J. (2012). A Library of cortical morphology analysis tools to study development, aging and genetics of cerebral cortex. Neuroinformatics 10, 81–96. 10.1007/s12021-011-9127-9 21698393 PMC3471145

[B34] Koepsell T. Monsell S. (2011). Characterizing individuals who revert from mild cognitive impairment to normal or near-normal cognition. Alzheimer's Dement. 7:S539. 10.1016/j.jalz.2011.05.152023019264

[B35] Kriegeskorte N. Simmons W. K. Bellgowan P. S. Baker C. I. (2009). Circular analysis in systems neuroscience: the dangers of double dipping. Nat. Neurosci. 12, 535–540. 10.1038/nn.2303 19396166 PMC2841687

[B36] Lebedev A. V. Westman E. Van Westen G. J. Kramberger M. G. Lundervold A. Aarsland D.. (2014). Random forest ensembles for detection and prediction of Alzheimer's disease with a good between-cohort robustness. Neuroimage Clin. 6, 115–125. 10.1016/j.nicl.2014.08.023 25379423 PMC4215532

[B37] Lebedeva A. K. Westman E. Borza T. Beyer M. K. Engedal K. Aarsland D.. (2017). MRI-based classification models in prediction of mild cognitive impairment and dementia in late-life depression. Front. Aging Neurosci. 9:13. 10.3389/fnagi.2017.00013 28210220 PMC5288688

[B38] Li Y. Wang Y. P. Wu G. R. Shi F. Zhou L. P. Lin W. L.. (2012). Discriminant analysis of longitudinal cortical thickness changes in Alzheimer's disease using dynamic and network features. Neurobiol. Aging 33, 427.e15-30. 10.1016/j.neurobiolaging.2010.11.008 21272960 PMC3086988

[B39] Liu M. Zhang J. Yap P. T. Shen D. (2017). View-aligned hypergraph learning for Alzheimer's disease diagnosis with incomplete multi-modality data. Med. Image Anal. 36, 123–134. 10.1016/j.media.2016.11.002 27898305 PMC5239753

[B40] Liu S. Q. Liu S. D. Cai W. D. Che H. Y. Pujol S. Kikinis R.. (2015). Multimodal Neuroimaging feature learning for multiclass diagnosis of Alzheimer's disease. IEEE TBE 62, 1132–1140. 10.1109/TBME.2014.2372011 25423647 PMC4394860

[B41] Liu T. Sachdev P. S. Lipnicki D. M. Jiang J. Geng G. Zhu W.. (2013). Limited relationships between two-year changes in sulcal morphology and other common neuroimaging indices in the elderly. Neuroimage 83, 12–17. 10.1016/j.neuroimage.2013.06.058 23800792

[B42] Lopez O. L. Becker J. T. Chang Y. F. Sweet R. A. DeKosky S. T. Gach M. H.. (2012). Incidence of mild cognitive impairment in the pittsburgh cardiovascular health study-cognition study. Neurology 79, 1599–1606. 10.1212/WNL.0b013e31826e25f0 23019262 PMC3475628

[B43] Maclin R. Opitz D. (1999). Popular ensemble methods: an empirical study. J. Artif. Intell. Res. 11, 169–198.

[B44] Macqueen J. (1967). Some methods for classification and analysis of multivariate observations, in Proceedings of Berkeley Symposium on Mathematical Statistics and Probability (Berkeley, CA: University of California Press), 281–297.

[B45] Mayo C. D. Mazerolle E. L. Ritchie L. Fisk J. D. Gawryluk J. R. Alzheimer's Disease Neuroimaging Initiative. (2017). Longitudinal changes in microstructural white matter metrics in Alzheimer's disease. Neuroimage Clin. 13, 330–338. 10.1016/j.nicl.2016.12.012 28066707 PMC5200876

[B46] McEvoy L. K. Holland D. Hagler D. J. Jr. Fennema-Notestine C. Brewer J. B. Dale A. M.. (2011). Mild cognitive impairment: baseline and longitudinal structural MR imaging measures improve predictive prognosis. Radiology 259, 834–843. 10.1148/radiol.11101975 21471273 PMC3099042

[B47] Min R. Wu G. Cheng J. Wang Q. Shen D. Alzheimer's Disease Neuroimaging Initiative. (2014). Multi-atlas based representations for Alzheimer's disease diagnosis. Hum. Brain Mapp. 35, 5052–5070. 10.1002/hbm.22531 24753060 PMC4169318

[B48] Misra C. Fan Y. Davatzikos C. (2009). Baseline and longitudinal patterns of brain atrophy in MCI patients, and their use in prediction of short-term conversion to AD: results from ADNI. Neuroimage 44, 1415–1422. 10.1016/j.neuroimage.2008.10.031 19027862 PMC2648825

[B49] Moradi E. Pepe A. Gaser C. Huttunen H. Tohka J. Alzheimer's Disease Neuroimaging I. (2015). Machine learning framework for early MRI-based Alzheimer's conversion prediction in MCI subjects. Neuroimage 104, 398–412. 10.1016/j.neuroimage.2014.10.002 25312773 PMC5957071

[B50] Mwangi B. Tian T. S. Soares J. C. (2014). A review of feature reduction techniques in neuroimaging. Neuroinformatics 12, 229–244. 10.1007/s12021-013-9204-3 24013948 PMC4040248

[B51] Pandya S. Y. Clem M. A. Silva L. M. Woon F. L. (2016). Does mild cognitive impairment always lead to dementia? A review. J. Neurol. Sci. 369, 57–62. 10.1016/j.jns.2016.07.055 27653867

[B52] Patenaude B. Smith S. M. Kennedy D. N. Jenkinson M. (2011). A Bayesian model of shape and appearance for subcortical brain segmentation. Neuroimage 56, 907–922. 10.1016/j.neuroimage.2011.02.046 21352927 PMC3417233

[B53] Pedregosa F. Varoquaux G. Gramfort A. Michel V. Thirion B. Grisel O. (2011). Scikit-learn: machine learning in python. J. Mach. Learn. Res. 12, 2825–2830.

[B54] Penttilae J. Paillere-Martinot M. L. Martinot J. L. Ringuenet D. Wessa M. Houenou J. (2009). Cortical folding in patients with bipolar disorder or unipolar depression. J. Psychiatry Neurosci. 34, 127–135. 19270763 PMC2647564

[B55] Pereira F. Mitchell T. Botvinick M. (2009). Machine learning classifiers and fMRI: a tutorial overview. Neuroimage 45, S199–S209. 10.1016/j.neuroimage.2008.11.007 19070668 PMC2892746

[B56] Petersen R. C. (2004). Mild cognitive impairment as a diagnostic entity. J. Intern. Med. 256, 183–194. 10.1111/j.1365-2796.2004.01388.x 15324362

[B57] Petersen R. C. (2010). Does the source of subjects matter? Absolutely! Neurology 74, 1754–1755. 10.1212/WNL.0b013e3181e533e7 20484685

[B58] Reuter M. Fischl B. (2011). Avoiding asymmetry-induced bias in longitudinal image processing. Neuroimage 57, 19–21. 10.1016/j.neuroimage.2011.02.076 21376812 PMC3260043

[B59] Reuter M. Schmansky N. J. Rosas H. D. Fischl B. (2012). Within-subject template estimation for unbiased longitudinal image analysis. Neuroimage 61, 1402–1418. 10.1016/j.neuroimage.2012.02.084 22430496 PMC3389460

[B60] Risacher S. L. Shen L. West J. D. Kim S. McDonald B. C. Beckett L. A.. (2010). Longitudinal MRI atrophy biomarkers: relationship to conversion in the ADNI cohort. Neurobiol. Aging 31, 1401–1418. 10.1016/j.neurobiolaging.2010.04.029 20620664 PMC2904350

[B61] Rivière D. Geffroy D. Denghien I. Souedet N. Cointepas Y. (2009). BrainVISA: an extensible software environment for sharing multimodal neuroimaging data and processing tools. Neuroimage 47, S163–S163. 10.1016/S1053-8119(09)71720-3

[B62] Riviere D. Mangin J. F. Papadopoulos-Orfanos D. Martinez J. M. Frouin V. Regis J. (2002). Automatic recognition of cortical sulci of the human brain using a congregation of neural networks. Med. Image Anal. 6, 77–92. 10.1016/S1361-8415(02)00052-X 12044997

[B63] Roberts R. O. Knopman D. S. Mielke M. M. Cha R. H. Pankratz V. S. Christianson T. J. H.. (2014). Higher risk of progression to dementia in mild cognitive impairment cases who revert to normal. Neurology 82, 317–325. 10.1212/WNL.0000000000000055 24353333 PMC3929198

[B64] Rosen W. G. Mohs R. C. Davis K. L. (1984). A new rating scale for Alzheimer's disease. Am. J. Psychiatry 141, 1356–1364. 10.1176/ajp.141.11.1356 6496779

[B65] Rusinek H. De Santi S. Frid D. Tsui W. H. Tarshish C. Y. Convit A.. (2003). Regional brain atrophy rate predicts future cognitive decline: 6-year longitudinal MR imaging study of normal aging. Radiology 229, 691–696. 10.1148/radiol.2293021299 14657306

[B66] Sabuncu M. R. Desikan R. S. Sepulcre J. Yeo B. T. Liu H. Schmansky N. J.. (2011). The dynamics of cortical and hippocampal atrophy in Alzheimer disease. Arch. Neurol. 68, 1040–1048. 10.1001/archneurol.2011.167 21825241 PMC3248949

[B67] Sachdev P. S. Brodaty H. Reppermund S. Kochan N. A. Trollor J. N. Draper B.. (2010). The Sydney Memory and Ageing Study (MAS): methodology and baseline medical and neuropsychiatric characteristics of an elderly epidemiological non-demented cohort of Australians aged 70-90 years. Int. Psychogeriatr. 22, 1248–1264. 10.1017/S1041610210001067 20637138

[B68] Schoonheim M. M. Hulst H. E. Brandt R. B. Strik M. Wink A. M. Uitdehaag B. M. J.. (2015). Thalamus structure and function determine severity of cognitive impairment in multiple sclerosis. Neurology 84, 776–783. 10.1212/WNL.0000000000001285 25616483

[B69] Shao J. Myers N. Yang Q. Feng J. Plant C. Bohm C.. (2012). Prediction of Alzheimer's disease using individual structural connectivity networks. Neurobiol. Aging 33, 2756–2765. 10.1016/j.neurobiolaging.2012.01.017 22405045 PMC3778749

[B70] Shi F. Liu B. Zhou Y. Yu C. Jiang T. (2010). Hippocampal volume and asymmetry in mild cognitive impairment and Alzheimer's disease: meta-analyses of MRI studies. Hippocampus 19, 1055–1064. 10.1002/hipo.20573 19309039

[B71] Sun Z. Y. Riviere D. Poupon F. Regis J. Mangin J. F. (2007). Automatic inference of sulcus patterns using 3D moment invariants. Med. Image Comput. Comput. Assist. Interv. 4791, 515–522. 10.1007/978-3-540-75757-3_63 18051098

[B72] Tabert M. H. Manly J. J. Liu X. Pelton G. H. Rosenblum S. Jacobs M.. (2006). Neuropsychological prediction of conversion to Alzheimer disease in patients with mild cognitive impairment. Arch. Gen. Psychiatry 63, 916–924. 10.1001/archpsyc.63.8.916 16894068

[B73] Tohka J. Moradi E. Huttunen H. Alzheimer's Disease Neuroimaging Initiative. (2016). Comparison of feature selection techniques in machine learning for anatomical brain MRI in dementia. Neuroinformatics 14, 279–296. 10.1007/s12021-015-9292-3 26803769

[B74] Trojanowski J. Q. Vandeerstichele H. Korecka M. Clark C. M. Aisen P. S. Petersen R. C.. (2010). Update on the biomarker core of the Alzheimer's disease neuroimaging initiative subjects. Alzheimer's Dement. 6, 230–238. 10.1016/j.jalz.2010.03.008 20451871 PMC2867838

[B75] Trzepacz P. T. Yu P. Sun J. Schuh K. Case M. Witte M. M.. (2014). Comparison of neuroimaging modalities for the prediction of conversion from mild cognitive impairment to Alzheimer's dementia. Neurobiol. Aging 35, 143–151. 10.1016/j.neurobiolaging.2013.06.018 23954175

[B76] Vapnik V. N. (1995). The Nature of Statistical Learning Theory. New York, NY: Springer.

[B77] Walt S. V. D. Colbert S. C. Varoquaux G. (2011). The NumPy array: a structure for effcient numerical computation. Comput. Sci. Eng. 13, 22–30. 10.1109/MCSE.2011.37

[B78] Wee C.-Y. Yap P.-T. Li W. Denny K. Browndyke J. N. Potter G. G.. (2011). Enriched white matter connectivity networks for accurate identification of MCI patients. Neuroimage 54, 1812–1822. 10.1016/j.neuroimage.2010.10.026 20970508 PMC3008336

[B79] Wee C.-Y. Yap P.-T. Zhang D. Denny K. Browndyke J. N. Potter G. G.. (2012). Identification of MCI individuals using structural and functional connectivity networks. Neuroimage 59, 2045–2056. 10.1016/j.neuroimage.2011.10.015 22019883 PMC3254811

[B80] Wen W. Sachdev P. S. Li J. J. Chen X. H. Anstey K. J. (2009). White Matter Hyperintensities in the forties: their prevalence and topography in an epidemiological sample aged 44-48. Hum. Brain Mapp. 30, 1155–1167. 10.1002/hbm.20586 18465744 PMC6870596

[B81] Whitwell J. L. Petersen R. C. Negash S. Weigand S. D. Kantarci K. Ivnik R. J.. (2007). Patterns of atrophy differ among specific subtypes of mild cognitive impairment. Arch. Neurol. 64, 1130–1138. 10.1001/archneur.64.8.1130 17698703 PMC2735186

[B82] Winblad B. Palmer K. Kivipelto M. Jelic V. Fratiglioni L. Wahlund L. O.. (2004). Mild cognitive impairment - beyond controversies, towards a consensus: report of the International Working Group on Mild Cognitive Impairment. J. Intern. Med. 256, 240–246. 10.1111/j.1365-2796.2004.01380.x 15324367

[B83] Yun H. J. Kwak K. Lee J. M. (2015). Multimodal discrimination of Alzheimer's Disease based on regional cortical atrophy and hypometabolism. PLoS ONE 10:e0129250. 10.1371/journal.pone.0129250 26061669 PMC4463854

[B84] Zhang D. Shen D. Alzheimer's Disease Neuroimaging Initiative. (2012a). Predicting future clinical changes of MCI patients using longitudinal and multimodal biomarkers. PLoS ONE 7:e33182. 10.1371/journal.pone.0033182 22457741 PMC3310854

[B85] Zhang D. Wang Y. Zhou L. Yuan H. Shen D. (2011). Multimodal classification of Alzheimer's disease and mild cognitive impairment. Neuroimage 55, 856–867. 10.1016/j.neuroimage.2011.01.008 21236349 PMC3057360

[B86] Zhang H. B. Sachdev P. S. Wen W. Kochan N. A. Crawford J. D. Brodaty H.. (2012b). Gray matter atrophy patterns of mild cognitive impairment subtypes. J. Neurol. Sci. 315, 26–32. 10.1016/j.jns.2011.12.011 22280946

[B87] Zhang J. Liu M. An L. Gao Y. Shen D. (2017). Alzheimer's disease diagnosis using landmark-based features from longitudinal structural mr images. IEEE J. Biomed. Health Informat. [Epub ahead of print]. 10.1109/JBHI.2017.2704614 28534798 PMC5685894

[B88] Zhou J. Liu J. Narayan V. A. Ye J. Alzheimer's Disease Neuroimaging Initiative. (2013). Modeling disease progression via multi-task learning. Neuroimage 78, 233–248. 10.1016/j.neuroimage.2013.03.073 23583359

